# The impact of exercise intervention on social interaction in children with autism: a network meta-analysis

**DOI:** 10.3389/fpubh.2024.1399642

**Published:** 2024-08-14

**Authors:** Yaoqi Hou, Zhuo Song, Jiaqi Deng, Xiangqin Song

**Affiliations:** College of Physical Education and Sports, Beijing Normal University, Beijing, China

**Keywords:** autism spectrum disorders, physical activity, network meta-analysis, social functioning, exercise

## Abstract

**Background:**

Extensive research has documented the positive impacts of physical activity on children and adolescents with Autism Spectrum Disorders (ASD). However, the specific benefits of various sports on the social functioning of children with ASD remain ambiguous. This study aims to employ a network meta-analysis to investigate the effects of different sports on the social functioning of children and adolescents with ASD and to establish a ranking of their effectiveness.

**Methods:**

This study conducted a comprehensive online search across Web of Science, PubMed, Cochrane, and Embase databases for randomized controlled trials and quasi-experimental studies focusing on social functioning outcomes. Data were synthesized using a Bayesian framework.

**Results:**

Sixteen relevant studies encompassing 560 participants were included. According to Cohen’s classification, mini-basketball (SMD = 0.84, 95% CI: 0.46, 1.20), SPARK (SMD = 0.88, 95% CI: 0.06, 1.70), and Karate (SMD = 1.10, 95% CI: 0.27, 2.00) demonstrated high effect sizes, with Karate identified as the most effective intervention. Conversely, Combined Exercise and Nei Yang Gong interventions exhibited the least significant effects, falling below small effect sizes.

**Conclusion:**

Physical activity interventions have been shown to enhance social functioning in children and adolescents with ASD to varying extents, with Karate emerging as the most efficacious.

## 1 Introduction

As of 2020, the CDC reports that about 17% of children aged 3 to 17 have neurodevelopmental disorders. Among them, approximately 1 in 36 has autism spectrum disorder (ASD) (1). ASD is a neurodevelopmental condition influenced by genetic, environmental, and immunological factors. It is marked by deficits in social functioning, repetitive behaviors, restricted interests, and executive functioning impairments (2, 3). Social functioning deficits in children with ASD mainly affect language processing, non-verbal communication, and social cognition (4). These challenges hinder social interactions and extend to emotional regulation, self-care, and family dynamics (5, 6). For instance, children with ASD may struggle with verbal communication, non-verbal social cues like facial expressions and body language, and understanding others’ intentions and emotions. These limitations restrict personal development and increase the caregiving burden on families. Therefore, providing targeted support and interventions is crucial to improve social functioning, education, daily activities, and social engagement.

The primary interventions for autism spectrum disorder (ASD) include pharmacological and behavioral strategies aimed at mitigating core symptoms, enhancing social functioning, and reducing related symptoms (7–9). Pharmacological approaches can have long initiation periods, delayed outcomes, and potential adverse effects on overall health (10). Behavioral interventions for children with ASD include Sensory Integration Training (SIT) for sensory adaptation (11); the Denver Early Start Model (ESDM) for improving language and social skills (12) Naturalistic Developmental Behavioral Interventions (NDBI) (13) and Parent-Mediated Interventions (PMI) (14) which facilitate learning in daily activities. Cognitive Behavioral Therapy (CBT) has shown efficacy in emotion regulation (15); and Social Skills Training (SST) concentrates on enhancing social competencies (16). However, these methods often require significant time, financial resources, and expertise. For example, ESDM and NDBI need prolonged professional involvement, PMI demands extensive parental engagement, CBT may not suit children with limited cognitive abilities, and SST skills can be hard to apply in real-world settings. Conversely, exercise interventions improve physical health, enhance social interactions, reduce behavioral issues, and support neurodevelopment. Exercise-based approaches are more cost-effective, easier to integrate into daily routines, and less burdensome for families. Physical activities can also improve family interactions and emotional bonds, creating a more enjoyable learning environment for children with ASD. Thus, given the benefits and limitations of different strategies, exercise intervention stands out as a straightforward and viable alternative.

Lopez-Diaz (17) employed football as an intervention and observed significant enhancements in social skills, emotional responses, feelings, and interpersonal problem-solving abilities following a 17-week program with 13 children diagnosed with ASD, in comparison to baseline measurements. Similarly, diverse forms of physical activity, including Karate (18), judo (19), Aquatic sports (20), and Combined sports (21) have demonstrated substantial improvements in the social functioning of children with ASD. Although numerous studies have corroborated the positive effects of exercise on the social functioning of children with ASD, direct comparisons between different exercise interventions remain scarce. Traditional meta-analysis methods face inherent challenges when comparing the efficacy of various interventions. Network Meta-Analysis (NMA), as an innovative approach, can integrate multiple interventions within a single analysis, leveraging both direct and indirect evidence for comparison. The primary advantage of this method lies in its ability to rank the effectiveness of various interventions based on a unified outcome measure and to calculate the relative efficacy probabilities for each intervention. The benefits of NMA include increased precision in estimating treatment effects. Even when direct comparisons are sparse or unavailable, NMA can still utilize all available evidence for comparison and analysis (22). NMA provides a more comprehensive analysis and understanding of the relative efficacy of multiple interventions by integrating data from various sources to create a connected network of comparisons. The impact of physical activity on various symptoms of children and adolescents with ASD has been widely studied, but researchers cannot directly compare all possible activities. In such cases, NMA can be used to analyze and compare the effects of different exercise interventions. Additionally, NMA can identify potential inconsistencies in the evidence and provide more reliable and robust estimates of treatment effects (23). In this study, the NMA method was used to compare the relative efficacy of different physical activities in improving the social functioning of children and adolescents with autism spectrum disorder (ASD). The goal is to identify the optimal physical activity intervention program.

## 2 Methods

### 2.1 Protocol

This study was conducted in accordance with the Preferred Reporting Items for Systematic Reviews and Network Meta-Analyses (PRISMA-NMA) guidelines (24). The PRISMA checklist is available in Appendix 1.

### 2.2 Search strategy

**Figure 1:**
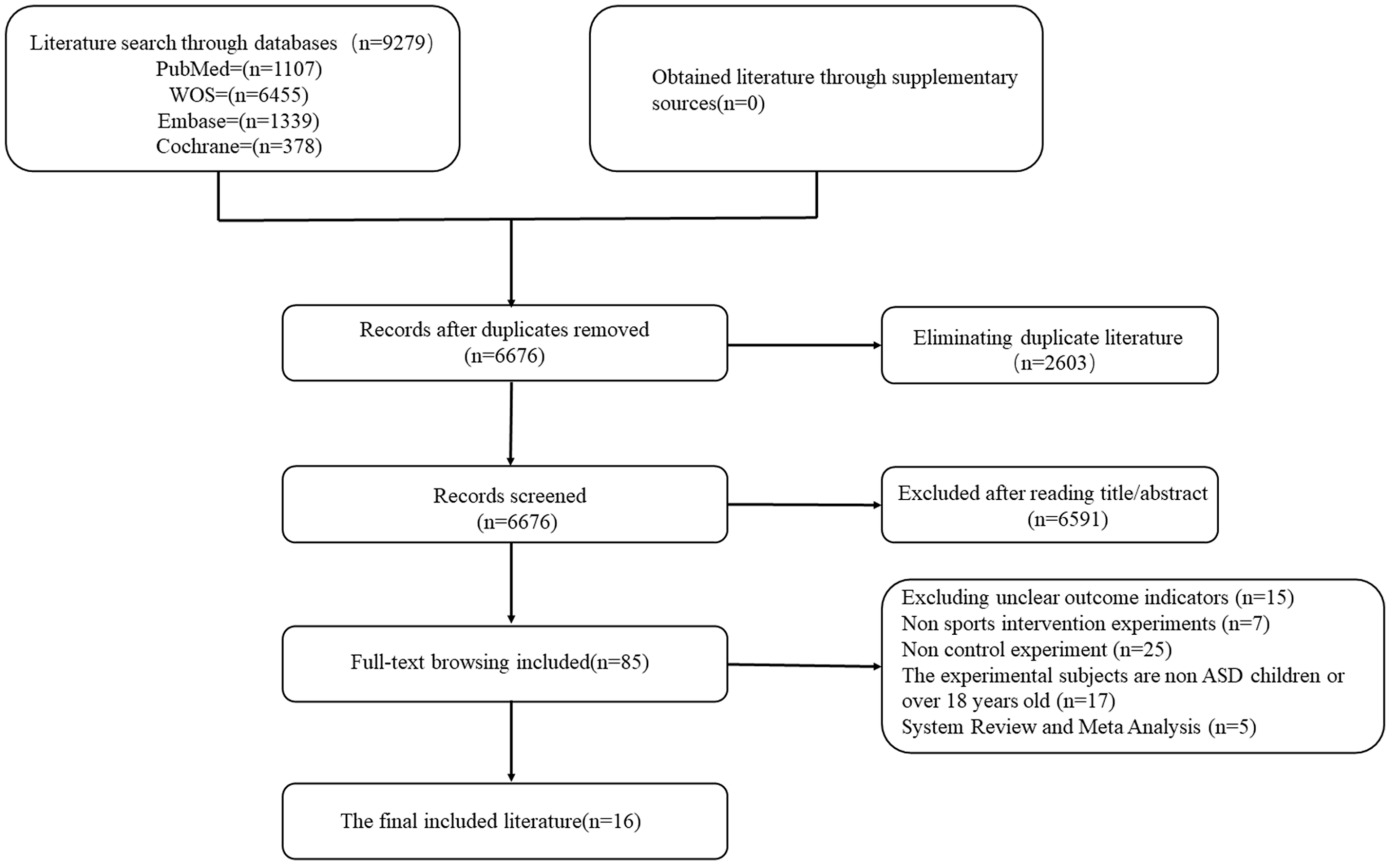
Flowchart of literature screening.

A comprehensive literature search was conducted across several databases, including The Cochrane Library, Embase, Web of Science, and PubMed, adhering to the “Patient Population, Intervention, Comparative, Outcome” (PICO) framework. This search utilized a combination of subject terms and free words, spanning from February 1, 2004, to March 1, 2024. To ensure the thoroughness of the literature included in this study, high-quality systematic reviews and meta-analyses relevant to the topic were also manually reviewed for potential inclusion. The process of the literature search is depicted in Figure 1, while the detailed search strategy is provided in Appendix 2.

### 2.3 Inclusion and exclusion criteria

Inclusion criteria: ① Experiment type: randomised controlled experiment or semi-randomised controlled experiment. ② Intervention subjects: children and adolescents who have been diagnosed with autism by authoritative institutions. ③ Intervention: including various forms of physical activities, in which the physical activities can be conducted in groups or individually, including but not limited to aerobic exercise, strength training, flexibility training, etc. Participants are required to engage in these activities on a regular basis to ensure the continuity and long-term effects of the intervention (minimum duration of 2 weeks, with a total number of interventions of not less than 8). The control group was set up without any sports interventions. These control conditions are intended to provide a baseline relative to the intervention group to assess the effectiveness of the physical activity intervention. ④ Outcome indicators: the scale or sub-dimension of the scale used to assess the social functioning of the test subjects, along with the requirement for relevant data that can be extracted, such as the standard deviation of the mean before and after the experiment.

Exclusion criteria: ① The outcome indicators are not clear, no extractable relevant data or not relevant to the selected outcome indicators. ② Conference papers, dissertations, reviews, case studies, uncontrolled studies. ③ Subjects suffering from multiple diseases at the same time.

### 2.4 Study selection and data extraction

Two researchers independently screened the titles and abstracts of the literature based on predefined inclusion and exclusion criteria. Afterward, they compared their results and merged their lists to identify disagreements. For inconsistent screenings, the researchers discussed each study to reach a consensus. If they could not agree, a third researcher reviewed the disputed studies and made the final decision. Studies with insufficient intervention descriptions or lacking mean and standard deviation data were also reviewed by a third researcher. After screening, relevant information from the included studies was extracted and compiled, encompassing: ① Basic paper information (author, nationality, publication year); ② Experimental design details (ASD diagnostic criteria, subject age range, average age, sample size of experimental and control groups, gender ratio); ③ Physical activity program specifics (activity type, intervention duration, frequency, control measures); ④ Executive and social function test scales, including pre- and post-intervention means and standard deviations; ⑤ Cochrane quality assessment indicators for the studies. Given that some scales indicated better outcomes with lower scores, while others indicated better outcomes with higher scores, effect sizes were adjusted for consistency. Specifically, for studies where lower scores denoted better outcomes, means and standard deviations were multiplied by −1 to align the direction of effect sizes with those studies where higher scores indicated better outcomes.

### 2.5 Study quality assessment

This study used the Cochrane Collaboration’s risk of bias tool to evaluate the quality of selected randomized controlled trials (RCTs). This tool assesses study design, conduct, and reporting quality to identify potential biases that might affect the study outcomes. The dimensions covered include random sequence generation, allocation concealment, blinding of participants and personnel, blinding of outcome assessment, completeness of outcome data, selective outcome reporting, and other potential biases. Evaluating these dimensions is crucial for identifying flaws in study design and execution, which can influence result interpretation.

### 2.6 Statistical analysis

The reticulated meta-analysis utilized R software (version 4.3.2) and the ‘Gemte’ package, in conjunction with the JAGS program and the Bayesian Markov Chain Monte Carlo (MCMC) algorithm, for a comprehensive analysis within the random effects model. This Bayesian approach utilized four Markov chains per model, setting 20,000 iterations with the first 5,000 for annealing. Model convergence was diagnosed using Potential Scale Reduction Factors (PSRF), with values close to 1 indicating satisfactory convergence. Effect sizes were estimated using the Standardized Mean Difference (SMD) and 95% Confidence Intervals (CI), addressing measurement variability across studies. Cohen’s classification was used for interpreting effect sizes: SMD ≥ |0.8| indicated a large effect, ≥ |0.5| to < |0.8| a medium effect, ≥ |0.2| to < |0.5| a small effect, and < |0.2| a negligible effect. The analysis began by mapping the network evidence, delineating direct and indirect comparisons. Direct comparisons were visualized with lines between dots, representing exercise intervention methods, where line thickness and dot size were proportional to the number of studies and sample sizes, respectively. For evidence networks forming a closed loop, a consistency test was conducted using the node-splitting method; a *p*-value greater than 0.05 indicated no significant inconsistency among interventions. Indirect comparisons were made through the reticulated meta-analysis framework for interventions lacking direct connections. A pairwise comparison among interventions was also performed to compute Surface Under the Cumulative Ranking (SUCRA) values, leading to a league table for intervention ranking. Interventions with SUCRA values closer to 1 were more effective, whereas those closer to 0 were less effective.

## 3 Results

### 3.1 Descriptions of included studies

**Figure 2:**
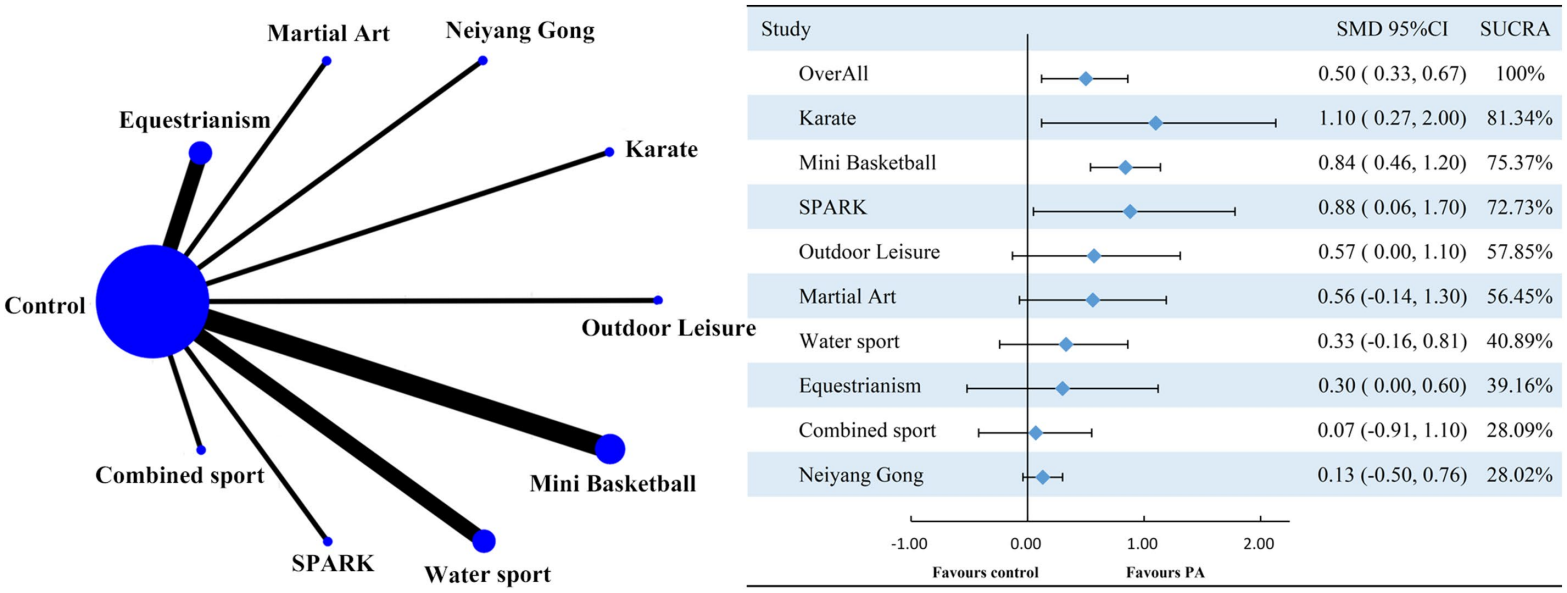
Network graph comparing the effect of various interventions on social function against the control group. PA represents physical activity; SMD stands for standardized mean difference; SUCRA denotes the surface under the cumulative ranking curve.

**Figure 3:**
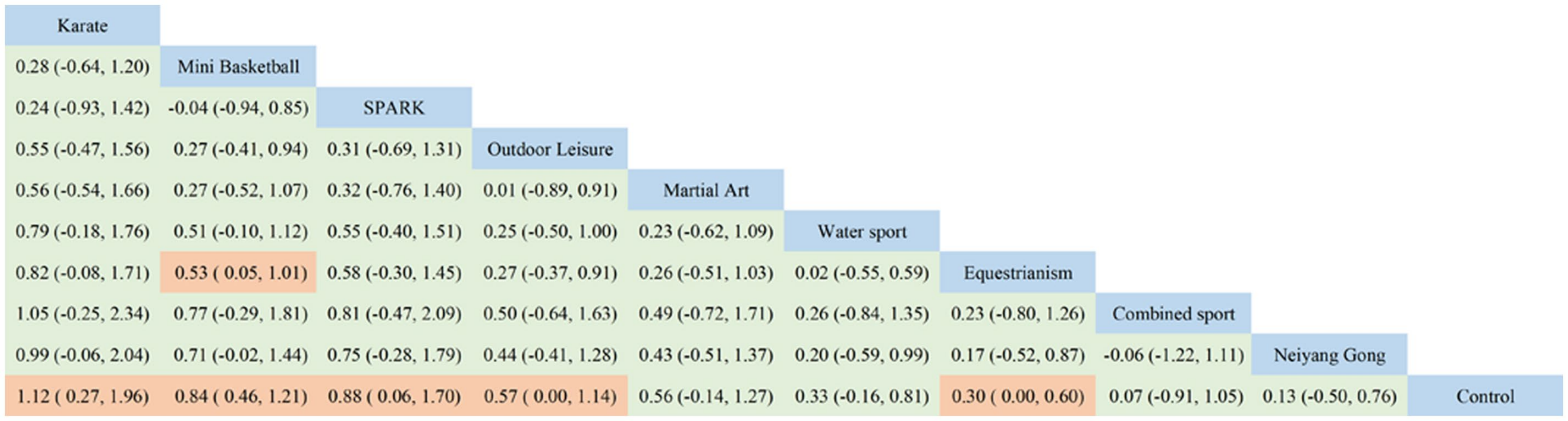
Ranking list of social function results analysis.

This study aimed to evaluate the effects of exercise interventions on the social functioning of children and adolescents with autism spectrum disorder (ASD) using a network meta-analysis (NMA). Due to the lack of closed-loop comparisons, traditional local inconsistency tests like the node-splitting method were not applicable. Thus, the consistency of direct and indirect comparison results could not be evaluated using standard methods. However, model convergence was assessed with the Potential Scale Reduction Factor (PSRF), and values close to 1 indicated robust convergence (Appendix 2, Figures 1, 2). The Deviance Information Criterion (DIC) difference between consistent and inconsistent models was less than 5, suggesting no significant discrepancy. This indicates that inconsistencies between direct and indirect evidence have minimal impact on the overall analysis results (Appendix 2, Figure 3).

A comprehensive search across multiple databases yielded 9,279 documents, all of which were imported into EndNote for de-duplication. This process eliminated 2,603 duplicates, leaving 6,676 relevant documents. Title and abstract screening excluded 6,591 papers as irrelevant, initially including 85 papers. Upon full-text review, 15 articles were excluded due to unclear outcome indicators, 7 due to non-exercise intervention types, 25 for lacking controlled experiments, 17 for involving subjects outside the specified age range or with conditions other than ASD, and 5 for being systematic reviews or meta-analyses. Ultimately, 16 pertinent articles were selected for inclusion, as depicted in Figure 1.

The data extraction form summarized key information, including the first author’s name, publication year, study country, subjects’ age range and mean age, gender distribution, experimental design, sample sizes of experimental and control groups, details of interventions, intervention duration, and scales used for outcome measurement. The included studies, published between 2009 and 2022, comprised 10 randomized controlled trials (RCTs) and 6 quasi-experimental designs, spanning 6 countries. The interventions varied, including Karate, Equestrianism, Water Sports, Neiyang Gong, Combined Sport, Mini Basketball, SPARK, and Martial Arts, with intervention durations ranging from 4 to 40 weeks and frequencies from once every 2 weeks to five times per week (detailed in Table 1).

**Table 1:** Basic characteristics of the included studies.

Study	Country	Sample age (years)	Female percentage (%)	Design	Sample size (E/C)	Intervention type		Intervention dose	Outcome indicator
						Experimental group	Control group		
Bass et al. (25)	United States	Mean:7.34 Range:5–10	14.71%	RCT	19/15	Equestrianism	Conventional therapy	60 min*1time*12 week	SRS-2
Borgi et al. (26)	Italy	Mean:8.6 Range:6–12	0.00%	RCT	15/13	Equestrianism	Conventional therapy	60 min*1time*25 week	VABS
Caputo et al. (27)	Italy	Mean:8 Range:6–13	34.62%	NRCT	13/13	Water Sports	Conventional therapy	45 min*2time*40 week	VABS
Chan et al. (28)	China	Mean:11.85 Range:6–17	10.00%	RCT	20/20	Neiyang Gong	Conventional therapy	60 min*2time*4 week	ATEC CCTT-T2
Gabriels et al. (29)	United States	Mean:10.25 Range:6–16	12.93%	RCT	58/58	Equestrianism	Conventional therapy	45 min*1time*10 week	VABS-2
Haghighi et al. (30)	Iran	Mean:8.57 Range:6–10	43.75%	RCT	8/8	Combined sport	Conventional therapy	60 min*3time*8 week	CPT
Cai et al. (31)	China	Mean:4.8 Range:3–6	16.13%	RCT	15/14	Mini Basketball	Conventional therapy	40 min*5time*12 week	SRS-2
Cai et al. (32)	China	Mean:4.91 Range:3–6	13.33%	NRCT	15/15	Mini Basketball	Conventional therapy	40 min*5time*12 week	SRS-2
Movahedi et al. (33)	Iran	Mean:9.3 Range:5–17	13.33%	RCT	13/13	Karate	Conventional therapy	30-90 min*4time*14 week	GARS-2
Najafabadi et al. (34)	Britain	Mean:7.58 Range:5–12	N/A	RCT	12/14	SPARK	Conventional therapy	40 min*3time*12 week	GARS-2
Pan et al. (35)	China	Mean:7.24 Range:6–9	0.00%	NRCT	8/8	Water Sports	Conventional therapy	90 min*2time*10 week	SSBS-2
Phung et al. (36)	United States	Mean:9.31 Range:8–11	17.65%	RCT	14/20	Martial Arts	Conventional therapy	45 min*2time*13 week	SSIS
Wang et al. (37)	China	Mean:4.91 Range:3–6	15.15%	NRCT	18/15	Mini Basketball	Conventional therapy	40 min*5time*12 week	SRS-2 CHEXI
Yang et al. (38)	China	Mean:4.85 Range:3–6	16.67%	RCT	15/15	Mini Basketball	Conventional therapy	40 min*5time*12 week	SRS-2
Zachor et al. (39)	Israel	Mean:5.3 Range:3–7	21.57%	NRCT	30/21	Outdoor Adventure	Conventional therapy	30 min*1time*13 week	SRS
Zanobini et al. (40)	Italy	Mean:5.56 Range:3–8	24.00%	NRCT	13/12	Water Sports	Conventional therapy	30 min*0.5time*24 week	SRS

For convenience, only the first author is given. N/A means not reported, and E/C means experimental group/control group. GARS-2: Gilliam autism rating scale, second edition; ATEC: autism treatment evaluation checklist; CPT: continuous performance test; SRS: social responsiveness scale; SRS-2: social responsiveness scale, second edition; SBS-2: school social behavior scales, second edition; SC: social communication; VABS: Vineland adaptive behavior scales; SSIS: social skills improvement system; SSIS-RS: social skills improvement system rating scales.

This meta-analysis scrutinized 16 studies on the social functioning of subjects with autism spectrum disorder (ASD). Although 10 studies achieved randomization, scores for allocation concealment and blinding of participants, personnel, and outcome assessment were notably low due to inherent challenges in exercise research. Consequently, many studies did not adhere to recommended blinding guidelines. The methodological quality scores were: one study scored 2 (low quality), six studies scored 3, seven studies scored 4, three studies scored 5, and one study scored 6 (highest quality). This distribution indicates that most studies were rated medium to high in methodological quality. Despite challenges with blinding, the scores suggest a generally positive assessment of the studies’ quality (Table 2).

**Table 2:** Quality evaluation of the included studies.

	Random sequence generation	Allocation concealment	Blinding of participants and personnel	Blinding of outcome assessment	Incomplete outcome data	Selective reporting	Other bias	Total
Bass (25)	1	0	0	0	1	0	1	3
Borgi (26)	1	0	0	0	1	1	1	4
Caputo (27)	0	0	0	1	1	1	1	4
Chan (28)	1	1	0	0	1	1	1	5
Gabriels (29)	1	0	0	0	1	1	1	4
Haghighi (30)	1	0	0	0	1	1	1	4
Cai et al. (31)	1	0	0	0	1	1	1	4
Cai et al. (32)	0	0	0	0	1	1	1	3
Movahedi (33)	1	0	0	1	1	1	1	5
Najafabadi (34)	1	0	0	1	1	1	1	5
Pan (35)	0	0	0	0	1	1	1	3
Phung (36)	1	0	0	0	1	1	1	4
Wang (37)	0	0	0	0	1	1	1	3
Yang (38)	1	1	0	1	1	1	1	6
Zachor (39)	0	0	0	0	1	0	1	2
Zanobini (40)	0	0	0	0	1	1	1	3

### 3.2 Network analysis results

This study included 16 papers with 560 participants: 286 in the experimental group and 274 in the control group. The studies on social functioning did not form a closed loop, comparing only exercise interventions with the control group, without inter-exercise comparisons. Network meta-analysis showed that all exercise interventions improved the social functioning of children with ASD, with a moderate overall effect size (SMD = 0.50, 95% CI: 0.33 to 0.67). Three interventions achieved a large effect size: mini-basketball (SMD = 0.84, 95% CI: 0.46 to 1.20, SUCRA: 75.37%), SPARK (SMD = 0.88, 95% CI: 0.06 to 1.70, SUCRA: 72.73%), and Karate (SMD = 1.10, 95% CI: 0.27 to 2.00, SUCRA: 81.34%). Two interventions had a medium effect size: Martial Arts (SMD = 0.56, 95% CI: −0.14 to 1.30, SUCRA: 56.45%) and Outdoor Leisure (SMD = 0.57, 95% CI: 0.00 to 1.10, SUCRA: 57.85%). Two slightly exceeded the small effect level: Equestrianism (SMD = 0.30, 95% CI: 0.00 to 0.60, SUCRA: 39.16%) and Water Sports (SMD = 0.33, 95% CI: −0.16 to 0.81, SUCRA: 40.89%). Two were below the small effect level: Combined Sports (SMD = 0.07, 95% CI: 0.00 to 0.60, SUCRA: 28.09%) and Neiyang Gong (SMD = 0.13, 95% CI: −0.50 to 0.76, SUCRA: 28.02%).

Based on the Surface Under the Cumulative Ranking (SUCRA) values (Figure 2) and cumulative probability plots (Appendix, Supplementary Figure 4), Karate was the most effective physical activity intervention for enhancing social functioning in children with autism spectrum disorder (ASD), with a SUCRA value of 81.34%. Mini basketball followed with a SUCRA value of 75.37%, and SPARK ranked third with a SUCRA value of 72.73%. All other sports interventions had SUCRA values below 70%. Ring inconsistency tests, which evaluate the concordance between direct and indirect effects in pairwise and multi-arm comparisons, were not applicable due to the absence of a closed loop. However, sensitivity analysis indicated that study quality variability did not affect the reliability of the results. Adjusted funnel plots revealed no significant publication bias (Appendix 2, Supplementary Figure 5). A meta-regression analysis, incorporating variables such as year of inclusion, subjects’ age, male-to-female ratio, weekly frequency of physical activity interventions, total and single intervention durations, and overall intervention period, showed no significant moderating effects. This supports the validity of the regression hypothesis. The meta-regression results are detailed in Appendix 2, Supplementary Figures 6, 7.

The presence of wide confidence intervals for certain interventions in this study can be attributed to two primary factors. Firstly, the process of selecting subjects specifically within the group of children with autism spectrum disorder (ASD) may have introduced limitations, directly impacting the precision of the effect estimates and consequently leading to broader confidence intervals. Secondly, the issue of network sparsity within this field of research underscores the challenge in directly comparing evidence of intervention effects. This scarcity of direct comparisons increases the uncertainty surrounding the estimates, further contributing to the expansion of the confidence intervals.

## 4 Discussion

Children with ASD participate less in physical activities and have lower physical fitness and motor competence than their typically developing peers (41). Research indicates that social functioning deficits in children with ASD may be linked to insufficient physical activity (42), Chonchaiya et al. found that a sedentary lifestyle and lack of exercise further decrease social skills in this population (43), Thelen’s ‘Embodied Cognition’ theory emphasizes the connection between socialization, communication, and movement, suggesting that sports engagement coordinates the brain’s social communication and motor systems, thus enhancing social functioning (44).

Exercise enhances executive and social functioning and reduces symptoms in children with autism spectrum disorder (ASD). Levante et al. found that regular physical activity improved social functioning and reduced repetitive behaviors in children with ASD (45), A controlled trial by Phung et al. showed that martial arts intervention significantly improved socially positive behaviors in children with ASD (36). Another study reported improvements in social functioning, emotion management, and executive functioning after 48 weeks of exercise intervention (46). Despite growing research on exercise effects on children with ASD, cross-sectional comparisons between different sports are lacking. This network meta-analysis indicates significant differences in social functioning improvement across various sports activities, providing preliminary evidence for future exercise intervention design. Karate showed the largest standardized mean difference (SMD), suggesting substantial improvement. However, wide confidence intervals and high uncertainty in Karate’s effect estimates may result from variations in sample sizes and intervention intensity or methodology. The SUCRA values indicated Karate ranked first among interventions, with an 81.34% probability of being the most effective. Mini Basketball and SPARK also yielded positive outcomes. Notably, Mini Basketball, with a narrower confidence interval (CI), more included studies, and a larger sample size, showed greater stability and reliability in results, with a SUCRA value of 75.37%.

Karate significantly improves the social functioning of children with ASD due to its structured environment and discipline. The regular, repetitive training helps establish a sense of security and predictability, reducing anxiety. Karate emphasizes etiquette and respect, enhancing social skills and self-discipline. Interactions with coaches and peers in a controlled environment allow children to practice social skills (47). Karate classes begin with etiquette practices like bowing and greetings, teaching respect and basic social manners, which children with ASD can apply in daily life. The physical contact and movements in karate improve coordination and balance (48). Training includes kicks, punches, and defenses, enhancing muscle strength, flexibility, and reaction speed, which translates to better daily life abilities and increased confidence. Karate’s goal-oriented approach, requiring mastery of specific skills for advancement, helps children set and achieve goals, enhancing their sense of accomplishment and self-efficacy (49). Group settings in karate provide a platform for social interaction, teaching teamwork and mutual support. Coaches provide positive feedback and guidance, building confidence and social skills. Karate benefits children with ASD physically, socially, and psychologically, making it a comprehensive intervention well-suited for them.

In a school environment, structured physical activities provide children with autism spectrum disorder (ASD) the opportunity to regularly engage in exercise, which helps improve their social interactions and overall well-being. Programs such as karate, mini-basketball, and SPARK (Sports, Play, and Active Recreation for Kids) can teach discipline, self-control, teamwork, and coordination skills. The advantages of implementing these programs in schools include students participating in activities within a familiar environment, teachers being able to integrate physical activities into the daily curriculum, and promoting continuous engagement and progress. However, limitations in the school environment include time and resource constraints, the need for additional teacher training to effectively implement these programs, and the potential lack of appropriate facilities and equipment (50). In community centers, sports programs can create an inclusive space for ASD children, encouraging their participation in physical activities and promoting social and physical fitness. Activities such as horseback riding, aquatic sports, and comprehensive martial arts programs are common and can improve ASD children’s balance, emotional regulation, sensory integration, and motor skill development. The benefits of implementing these programs in community centers include offering a wider and more diverse range of activities, flexible scheduling, and forming a supportive network through the involvement of parents and caregivers, which further promotes children’s progress. However, limitations of community centers include the need to coordinate with local healthcare providers, therapists, and schools to ensure a multidisciplinary approach, as well as higher demands for resources and facilities, which may pose practical challenges (51). Overall, implementing these sports programs in different environments can provide multifaceted support and development opportunities for ASD children, but overcoming respective limitations is essential to ensure the effectiveness and sustainability of these interventions.

Despite the significant benefits of exercise interventions for children with ASD, existing literature has limitations. Most studies focus on a single exercise intervention, lacking direct comparisons between different programs. This limits understanding of the most effective exercise forms. Additionally, there is a shortage of high-quality, large-sample randomized controlled trials (RCTs), undermining the generalizability and reliability of findings. Many studies have small sample sizes and lack diversity, failing to represent the broader ASD population. Methodologically, most studies employ short-term interventions and follow-ups, making it difficult to assess long-term effects and sustainability. Future research should focus on larger, more representative samples and diverse methodologies to enhance findings’ generalizability and reliability. Innovative designs, like multi-arm RCTs and long-term follow-up studies, are needed to evaluate long-term effects and sustainability. It is crucial to explore different intensities, frequencies, and durations of exercise interventions and develop personalized plans. Combining exercise with virtual reality (VR) technology could enhance motor skills and social interaction abilities in children with ASD, offering a promising research direction. Specific methods could include several weekly exercise interventions, each lasting over 30 min, involving traditional sports (e.g., basketball and soccer) and VR simulations (e.g., virtual basketball games). VR can simulate real social scenarios, provide instant feedback, and offer progressive challenges, helping children improve motor skills and confidence in a safe environment. Recording exercise data through VR devices allows researchers to adjust the intensity and difficulty of exercises, ensuring effectiveness and adaptability. This multifaceted approach enhances intervention effectiveness and helps researchers understand the synergistic effects between different interventions, providing guidance for future research and practice.

## 5 Conclusion

This study explored the effects of different sports on social function in children with autism spectrum disorder (ASD) through a network meta-analysis. The results showed that physical activity intervention had a significant effect on improving social function in children with ASD, with karate, mini basketball, and SPARK interventions showing higher effect values. Karate has been identified as the most effective intervention measure. Future research should further explore the specific effects of different exercise programs to optimize exercise intervention programs for children with ASD.

## Data Availability

The original contributions presented in the study are included in the article/Supplementary material, further inquiries can be directed to the corresponding author.
