# Supplementary material for: The impact of exercise intervention on social interaction in children with autism: a network meta-analysis
Source: Front Public Health. 2024 Aug 14;12:1399642. doi: 10.3389/fpubh.2024.1399642 (PMC11349572; doi:10.3389/fpubh.2024.1399642)
Supplement: Supplementary file 2 [file Data_Sheet_2.docx]

Appendix 2

Supporting Documentation

Search strategy

Figure S1 Convergence diagnosis

Figure S2 Trajectory and density plots for consistency model testing of various intervention measures

Figure S3 Consistency model and inconsistency model

Figure S4 Cumulative Probability

Figure S5 Funnel plots of publication bias for outcome measures of included studies

Figure S6-S7 Regressive analysis

Search strategy

PubMed 1107

#1：((((Autism Spectrum Disorder[MeSH Terms]) OR (Autism Spectrum Disorders)) OR (Autistic Spectrum Disorder)) OR (Autistic Spectrum Disorders)) OR (Disorder, Autistic Spectrum)

#2：:((((Sports[MeSH Terms]) OR (((Sport[Title/Abstract]) OR (Athletics[Title/Abstract])) OR (Athletic[Title/Abstract]))) OR ((Exercise Therapy[MeSH Terms]) OR (((((((((((Remedial Exercise[Title/Abstract]) OR (Exercise, Remedial[Title/Abstract])) OR (Exercises, Remedial[Title/Abstract])) OR (Remedial Exercises[Title/Abstract])) OR (Therapy, Exercise[Title/Abstract])) OR (Exercise Therapies[Title/Abstract])) OR (Therapies, Exercise[Title/Abstract])) OR (Rehabilitation Exercise[Title/Abstract])) OR (Exercise, Rehabilitation[Title/Abstract])) OR (Exercises, Rehabilitation[Title/Abstract])) OR (Rehabilitation Exercises[Title/Abstract])))) OR ((Exercise[MeSH Terms]) OR ((((((((((((((((((((((((Exercises[Title/Abstract]) OR (Physical Activity[Title/Abstract])) OR (Activities, Physical[Title/Abstract])) OR (Activity, Physical[Title/Abstract])) OR (Physical Activities[Title/Abstract])) OR (Exercise, Physical[Title/Abstract])) OR (Exercises, Physical[Title/Abstract])) OR (Physical Exercise[Title/Abstract])) OR (Physical Exercises[Title/Abstract])) OR (Acute Exercise[Title/Abstract])) OR (Acute Exercises[Title/Abstract])) OR (Exercise, Acute[Title/Abstract])) OR (Exercises, Acute[Title/Abstract])) OR (Exercise, Isometric[Title/Abstract])) OR (Exercises, Isometric[Title/Abstract])) OR (Isometric Exercises[Title/Abstract])) OR (Isometric Exercise[Title/Abstract])) OR (Exercise, Aerobic[Title/Abstract])) OR (Exercise, Aerobic[Title/Abstract])) OR (Aerobic Exercise[Title/Abstract])) OR (Aerobic Exercises[Title/Abstract])) OR (Exercises, Aerobic[Title/Abstract])) OR (Exercise Training[Title/Abstract])) OR (Trainings, Exercise[Title/Abstract])))) OR (((((((((((((((((((((((((((((((((((((((((((Baseball[Title/Abstract]) OR (Basketball[Title/Abstract])) OR (Bicycling[Title/Abstract])) OR (Boxing[Title/Abstract])) OR (Cricket Sport[Title/Abstract])) OR (Football[Title/Abstract])) OR (Golf[Title/Abstract])) OR (Gymnastics[Title/Abstract])) OR (Hockey[Title/Abstract])) OR (Martial Arts[Title/Abstract])) OR (Mountaineering[Title/Abstract])) OR (Racquet[Title/Abstract])) OR (Return[Title/Abstract])) OR (Sport[Title/Abstract])) OR (Sports[Title/Abstract])) OR (Rugby[Title/Abstract])) OR (Running[Title/Abstract])) OR (Skating[Title/Abstract])) OR (Snow Sports[Title/Abstract])) OR (Soccer[Title/Abstract])) OR (train[Title/Abstract])) OR (fitness[Title/Abstract])) OR (aerobic[Title/Abstract])) OR (walking[Title/Abstract])) OR (high intensity interval[Title/Abstract])) OR (resistance[Title/Abstract])) OR (core stability[Title/Abstract])) OR (dance[Title/Abstract])) OR (breathing exercise[Title/Abstract])) OR (virtual reality exercise[Title/Abstract])) OR (whole body vibration exercise[Title/Abstract])) OR (stretching[Title/Abstract])) OR (body ⁃ mind exercise[Title/Abstract])) OR (Yoga[Title/Abstract])) OR (pilates[Title/Abstract])) OR (Tai Chi[Title/Abstract])) OR (Taijiquan[Title/Abstract])) OR (Health Qigong[Title/Abstract])) OR (Yijinjing[Title/Abstract])) OR (Wuqinxi[Title/Abstract])) OR (Liuzijue[Title/Abstract])) OR (Baduanjin[Title/Abstract])) OR (multicomponent exercise[Title/Abstract]))

#3：((((((((((((((((((((((((Child[MeSH Terms]) OR (Adolescent[MeSH Terms])) OR (Young Adult[MeSH Terms])) OR (Minor[MeSH Terms])) OR (Adolescen[Title/Abstract])) OR (Teen[Title/Abstract])) OR (Teenager[Title/Abstract])) OR (Youth[Title/Abstract])) OR (Minors[Title/Abstract])) OR (Minor[Title/Abstract])) OR (Child[Title/Abstract])) OR (Child[Title/Abstract])) OR (Kid[Title/Abstract])) OR (Kids[Title/Abstract])) OR (Girl[Title/Abstract])) OR (Boy[Title/Abstract])) OR (Under age[Title/Abstract])) OR (Underage[Title/Abstract])) OR (Young people[Title/Abstract])) OR (young person[Title/Abstract])) OR (Pubescen[Title/Abstract])) OR (Young Adult[Title/Abstract])) OR (School age[Title/Abstract])) OR (Preschool[Title/Abstract])) OR (Student[Title/Abstract])

#4：#1 AND #2 AND #3

WOS 6455

#1：((((TS=(Autism Spectrum Disorder)) OR TS=(Autism Spectrum Disorders)) OR TS=(Autistic Spectrum Disorder)) OR TS=(Autistic Spectrum Disorders)) OR TS=(Disorder, Autistic Spectrum)

#2：(TS=(Equine-Assisted Therapy)) OR (TS=(Animal Assisted Therapy)) OR (TS=(Baseball)) OR (TS=(Basketball)) OR (TS=(Bicycling)) OR (TS=(Boxing)) OR (TS=(Cricket Sport)) OR (TS=(Football)) OR (TS=(Golf)) OR (TS=(Gymnastics)) OR (TS=(Hockey)) OR (TS=(Martial Arts)) OR (TS=(Mountaineering)) OR (TS=(Racquet Sports)) OR (TS=(Return to Sport)) OR (TS=(Rugby)) OR (TS=(Running)) OR (TS=(Skating)) OR (TS=(Snow Sports)) OR (TS=(Soccer)) OR (TS=(train)) OR (TS=(fitness)) OR (TS=(aerobic)) OR (TS=(walking)) OR (TS=(high intensity interval)) OR (TS=(resistance)) OR (TS=(core stability)) OR (TS=(dance)) OR (TS=(breathing exercise)) OR (TS=(virtual reality exercise)) OR (TS=(whole body vibration exercise)) OR (TS=(stretching)) OR (TS=(body ⁃ mind exercise)) OR (TS=(Yoga)) OR (TS=(pilates)) OR (TS=(Tai Chi)) OR (TS=(Taijiquan)) OR (TS=(Health Qigong)) OR (TS=(Yijinjing)) OR (TS=(Wuqinxi)) OR (TS=(Liuzijue)) OR (TS=(Baduanjin)) OR (TS=(multicomponent exercise)) OR (((TS=(Sports)) OR TS=(Sport)) OR TS=(Athletics)) OR TS=(Athletic)OR (((((((((((TS=(Exercise Therapy)) OR TS=(Remedial Exercise)) OR TS=(Exercise, Remedial)) OR TS=(Exercises, Remedial)) OR TS=(Remedial Exercises)) OR TS=(Therapy, Exercise)) OR TS=(Exercise Therapies)) OR TS=(Therapies, Exercise)) OR TS=(Rehabilitation Exercise)) OR TS=(Exercise, Rehabilitation)) OR TS=(Exercises, Rehabilitation)) OR TS=(Rehabilitation Exercises)

OR (((((((((((((((((((((((((TS=(Exercise)) OR TS=(Exercises)) OR TS=(Physical Activity)) OR TS=(Activities, Physical)) OR TS=(Activity, Physical)) OR TS=(Physical Activities)) OR TS=(Exercise, Physical)) OR TS=(Exercises, Physical)) OR TS=(Physical Exercise)) OR TS=(Physical Exercises)) OR TS=( Acute Exercise)) OR TS=(Acute Exercises)) OR TS=(Exercise, Acute)) OR TS=(Exercises, Acute)) OR TS=(Exercise, Isometric)) OR TS=(Exercises, Isometric)) OR TS=(Isometric Exercises)) OR TS=(Isometric Exercise)) OR TS=(Exercise, Aerobic)) OR TS=(Aerobic Exercise)) OR TS=(Aerobic Exercises)) OR TS=(Exercises, Aerobic)) OR TS=(Exercise Training)) OR TS=(Exercise Trainings)) OR TS=(Training, Exercise)) OR TS=(Trainings, Exercise)

#3：((((((((((((((((((((((((TS=(Child)) OR TS=(Adolescent)) OR TS=(Young Adult )) OR TS=(Minor )) OR TS=(Adolescen)) OR TS=(Teen)) OR TS=(Teenager)) OR TS=(Youth)) OR TS=(Minors)) OR TS=(Minor)) OR TS=(Child)) OR TS=(Kid)) OR TS=(Kids)) OR TS=(Girl)) OR TS=(Boy)) OR TS=(Under age)) OR TS=(Underage)) OR TS=(Young people )) OR TS=(young person )) OR TS=(Prepubescen)) OR TS=(Pubescen)) OR TS=(Young Adult)) OR TS=(School age)) OR TS=(Preschool)) OR TS=(Student)

#4：#1 AND #2 AND #3

Cochrane 378

#1：Autism Spectrum Disorder

#2：(Autism Spectrum Disorders ):ab,ti,kw OR (Autistic Spectrum Disorder ):ab,ti,kw OR (Autistic Spectrum Disorders ):ab,ti,kw OR (Disorder, Autistic Spectrum ):ab,ti,kw

#3：Therapeutics

#4：(Therapeutic ):ab,ti,kw OR (Therapy ):ab,ti,kw OR (Therapies ):ab,ti,kw OR (Treatment ):ab,ti,kw OR (Treatments ):ab,ti,kw

#5：Sports

#6：(Sport ):ab,ti,kw OR (Athletics ):ab,ti,kw OR (Athletic ):ab,ti,kw

#7：Exercise

#8：(Exercises ):ab,ti,kw OR (Physical Activity ):ab,ti,kw OR (Activities, Physical ):ab,ti,kw OR (Activity, Physical ):ab,ti,kw OR (Physical Activities ):ab,ti,kw OR (Exercise, Physical ):ab,ti,kw OR (Exercises, Physical ):ab,ti,kw OR (Physical Exercise ):ab,ti,kw OR (Physical Exercises ):ab,ti,kw OR (Acute Exercise ):ab,ti,kw OR (Acute Exercises ):ab,ti,kw OR (Exercise, Acute ):ab,ti,kw OR (Exercises, Acute ):ab,ti,kw OR (Exercise, Isometric ):ab,ti,kw OR (Exercises, Isometric ):ab,ti,kw OR (Isometric Exercises ):ab,ti,kw OR (Isometric Exercise ):ab,ti,kw OR (Exercise, Aerobic ):ab,ti,kw OR (Aerobic Exercise ):ab,ti,kw OR (Aerobic Exercises ):ab,ti,kw OR (Exercises, Aerobic ):ab,ti,kw OR (Exercise Training ):ab,ti,kw OR (Exercise Trainings ):ab,ti,kw OR (Training, Exercise ):ab,ti,kw OR (Trainings, Exercise ):ab,ti,kw

#9：(Baseball ):ab,ti,kw OR (Basketball ):ab,ti,kw OR (Bicycling ):ab,ti,kw OR (Boxing ):ab,ti,kw OR (Cricket Sport ):ab,ti,kw OR (Football ):ab,ti,kw OR (Golf ):ab,ti,kw OR (Gymnastics ):ab,ti,kw OR (Hockey ):ab,ti,kw OR (Martial Arts ):ab,ti,kw OR (Mountaineering ):ab,ti,kw OR (Racquet Sports ):ab,ti,kw OR (Return to Sport ):ab,ti,kw OR (Rugby ):ab,ti,kw OR (Running ):ab,ti,kw OR (Skating ):ab,ti,kw OR (Snow Sports ):ab,ti,kw OR (Soccer ):ab,ti,kw OR (train ):ab,ti,kw OR (fitness ):ab,ti,kw OR (aerobic ):ab,ti,kw OR (walking ):ab,ti,kw OR (high intensity interval ):ab,ti,kw OR (resistance ):ab,ti,kw OR (core stability ):ab,ti,kw OR (dance ):ab,ti,kw OR (breathing exercise ):ab,ti,kw OR (virtual reality exercise ):ab,ti,kw OR (whole body vibration exercise ):ab,ti,kw OR (stretching ):ab,ti,kw OR (body mind exercise ):ab,ti,kw OR (Yoga ):ab,ti,kw OR (pilates ):ab,ti,kw OR (Tai Chi ):ab,ti,kw OR (Taijiquan ):ab,ti,kw OR (Health Qigong ):ab,ti,kw OR (Yijinjing ):ab,ti,kw OR (Wuqinxi ):ab,ti,kw OR (Liuzijue ):ab,ti,kw OR (Baduanjin ):ab,ti,kw OR (multicomponent exercise ):ab,ti,kw OR (Equine-Assisted Therapy ):ab,ti,kw OR (Animal Assisted Therapy ):ab,ti,kw

#10：Child OR Adolescent OR Young Adult OR Minor

#11：(Children ):ab,ti,kw OR (Children ):ab,ti,kw OR (Child ):ab,ti,kw OR (Adolescent ):ab,ti,kw OR (Young Adult ):ab,ti,kw OR (Minor ):ab,ti,kw OR (Adolescen ):ab,ti,kw OR (Teen ):ab,ti,kw OR (Teenager ):ab,ti,kw OR (Youth ):ab,ti,kw OR (Minors ):ab,ti,kw OR (Minor ):ab,ti,kw OR (Child ):ab,ti,kw OR (Kid ):ab,ti,kw OR (Girl ):ab,ti,kw OR (Boy ):ab,ti,kw OR (Under age ):ab,ti,kw OR (Underage ):ab,ti,kw OR (Young people):ab,ti,kw OR (young person ):ab,ti,kw OR (Prepubescen ):ab,ti,kw OR (Pubescen ):ab,ti,kw OR (Young Adult ):ab,ti,kw OR (School age ):ab,ti,kw OR (Preschool ):ab,ti,kw OR (Student ):ab,ti,kw

#12：#1 OR #2

#13：#3 OR #4 #5 OR #6 #7 OR #8 OR #9

#14：#10 OR #11

#15：#12 AND #13 AND #14

Embase 1339

#1：autism AND spectrum AND disorder

#2：'autism spectrum disorders':ab,ti OR 'autistic spectrum disorder':ab,ti OR 'autistic spectrum disorders':ab,ti OR 'disorder, autistic spectrum':ab,ti

#3：sports

#4：'sport':ab,ti OR 'athletics':ab,ti OR 'athletic':ab,ti

#5：exercise AND therapy

#6：'remedial exercise':ab,ti OR 'exercise, remedial':ab,ti OR 'exercises, remedial':ab,ti OR 'remedial exercises':ab,ti OR 'therapy, exercise':ab,ti OR 'exercise therapies':ab,ti OR 'therapies, exercise':ab,ti OR 'rehabilitation exercise':ab,ti OR 'exercise, rehabilitation':ab,ti OR 'exercises, rehabilitation':ab,ti OR 'rehabilitation exercises':ab,ti

#7：exercise

#8：'exercises':ab,ti OR 'physical activity':ab,ti OR 'activities, physical':ab,ti OR 'activity, physical':ab,ti OR 'physical activities':ab,ti OR 'exercise, physical':ab,ti OR 'exercises, physical':ab,ti OR 'physical exercise':ab,ti OR 'physical exercises':ab,ti OR 'acute exercise':ab,ti OR 'acute exercises':ab,ti OR 'exercise, acute':ab,ti OR 'exercises, acute':ab,ti OR 'exercise, isometric':ab,ti OR 'exercises, isometric':ab,ti OR 'isometric exercises':ab,ti OR 'isometric exercise':ab,ti OR 'exercise, aerobic':ab,ti OR 'aerobic exercise':ab,ti OR 'aerobic exercises':ab,ti OR 'exercises, aerobic':ab,ti OR 'exercise training':ab,ti OR 'exercise trainings':ab,ti OR 'training, exercise':ab,ti OR 'trainings, exercise':ab,ti

#9：'baseball':ab,ti OR 'basketball':ab,ti OR 'bicycling':ab,ti OR 'boxing':ab,ti OR 'cricket sport':ab,ti OR 'football':ab,ti OR 'golf':ab,ti OR 'gymnastics':ab,ti OR 'hockey':ab,ti OR 'martial arts':ab,ti OR 'mountaineering':ab,ti OR 'racquet sports':ab,ti OR 'return to sport':ab,ti OR 'rugby':ab,ti OR 'running':ab,ti OR 'skating':ab,ti OR 'snow sports':ab,ti OR 'soccer':ab,ti OR 'train':ab,ti OR 'fitness':ab,ti OR 'aerobic':ab,ti OR 'walking':ab,ti OR 'high intensity interval':ab,ti OR 'resistance':ab,ti OR 'core stability':ab,ti OR 'dance':ab,ti OR 'breathing exercise':ab,ti OR 'virtual reality exercise':ab,ti OR 'whole body vibration exercise':ab,ti OR 'stretching':ab,ti OR 'body mind exercise':ab,ti OR 'yoga':ab,ti OR 'pilates':ab,ti OR 'tai chi':ab,ti OR 'taijiquan':ab,ti OR 'health qigong':ab,ti OR 'yijinjing':ab,ti OR 'wuqinxi':ab,ti OR 'liuzijue':ab,ti OR 'baduanjin':ab,ti OR 'multicomponent exercise':ab,ti OR 'equine-assisted therapy':ab,ti OR 'animal assisted therapy':ab,ti

#10：'child'/exp OR child

#11：'children':ab,ti OR 'adolescent':ab,ti OR 'adolescen':ab,ti OR 'teen':ab,ti OR 'teenager':ab,ti OR 'youth':ab,ti OR 'minors':ab,ti OR 'minor':ab,ti OR 'child':ab,ti OR 'kid':ab,ti OR 'girl':ab,ti OR 'boy':ab,ti OR 'under age':ab,ti OR 'underage':ab,ti OR 'young people':ab,ti OR 'young person':ab,ti OR 'prepubescen':ab,ti OR 'pubescen':ab,ti OR 'young adult':ab,ti OR 'school age':ab,ti OR 'preschool':ab,ti OR 'student':ab,ti

#12：#1 OR #2

#13：#3 OR #4 #5 OR #6 #7 OR #8 OR #9

#14：#10 OR #11

#15：#12 AND #13 AND #14

**Figure 1** Convergence diagnosis


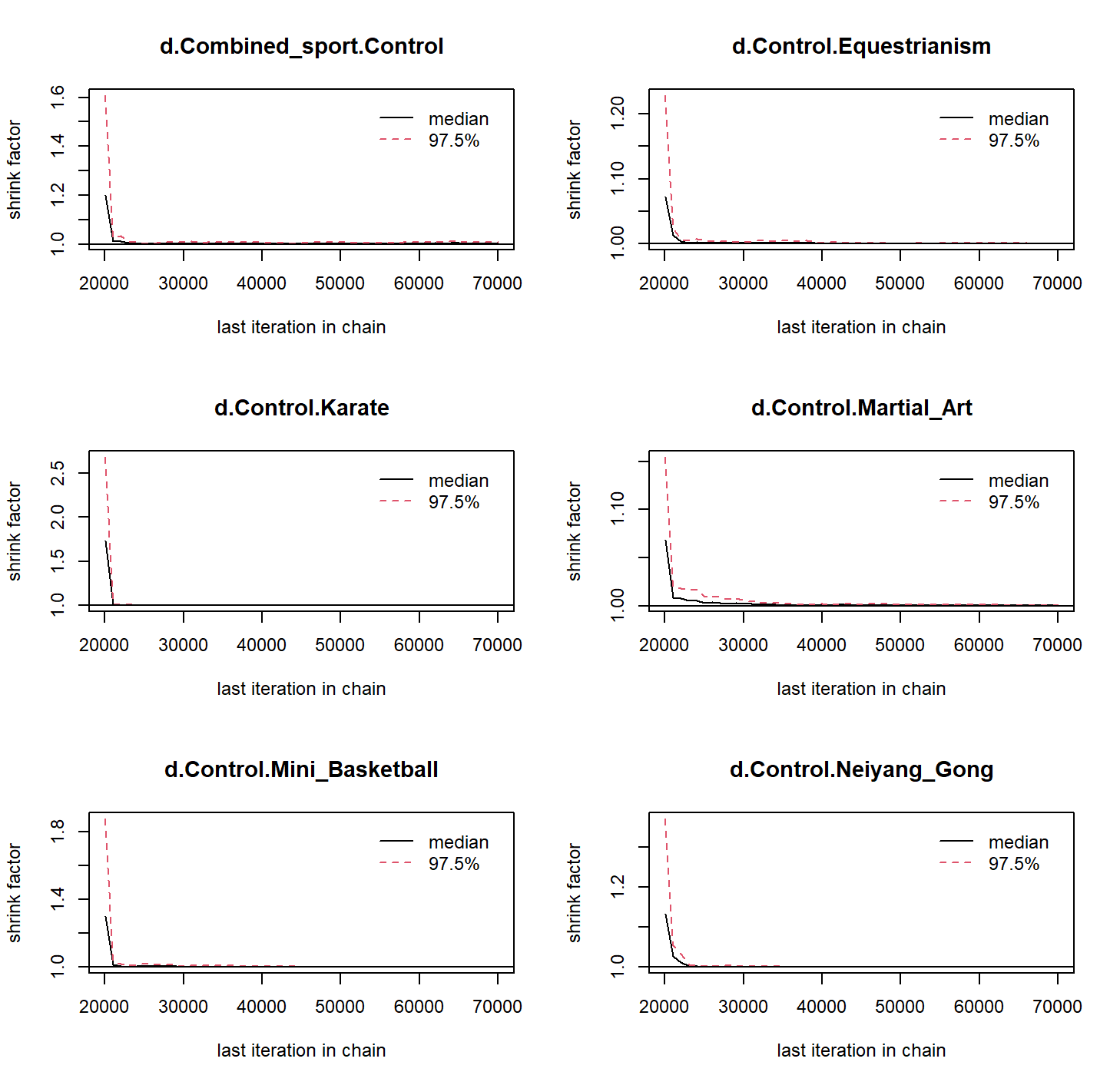


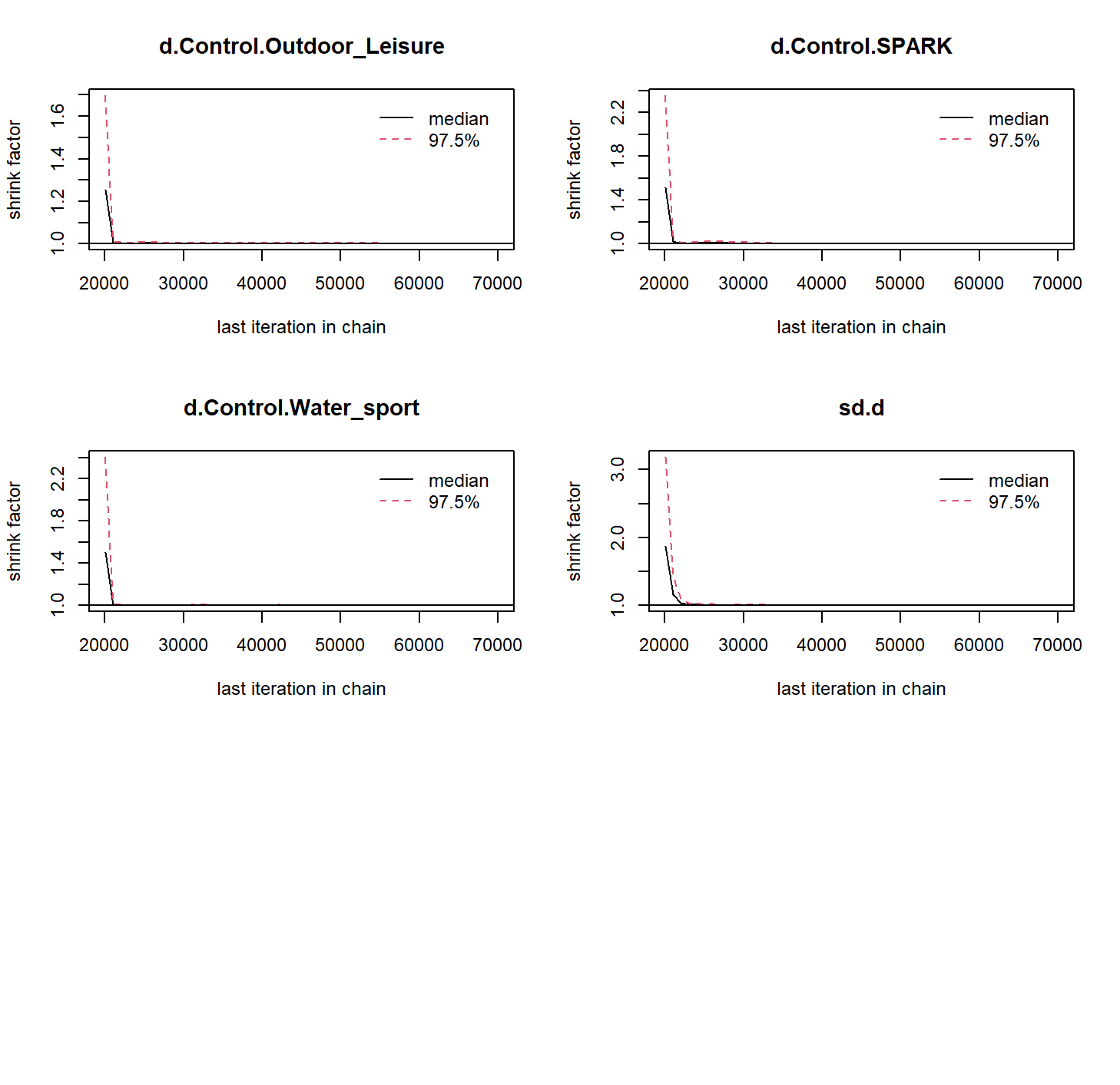


**Figure 2** Trajectory and density plots for consistency model testing of various intervention measures


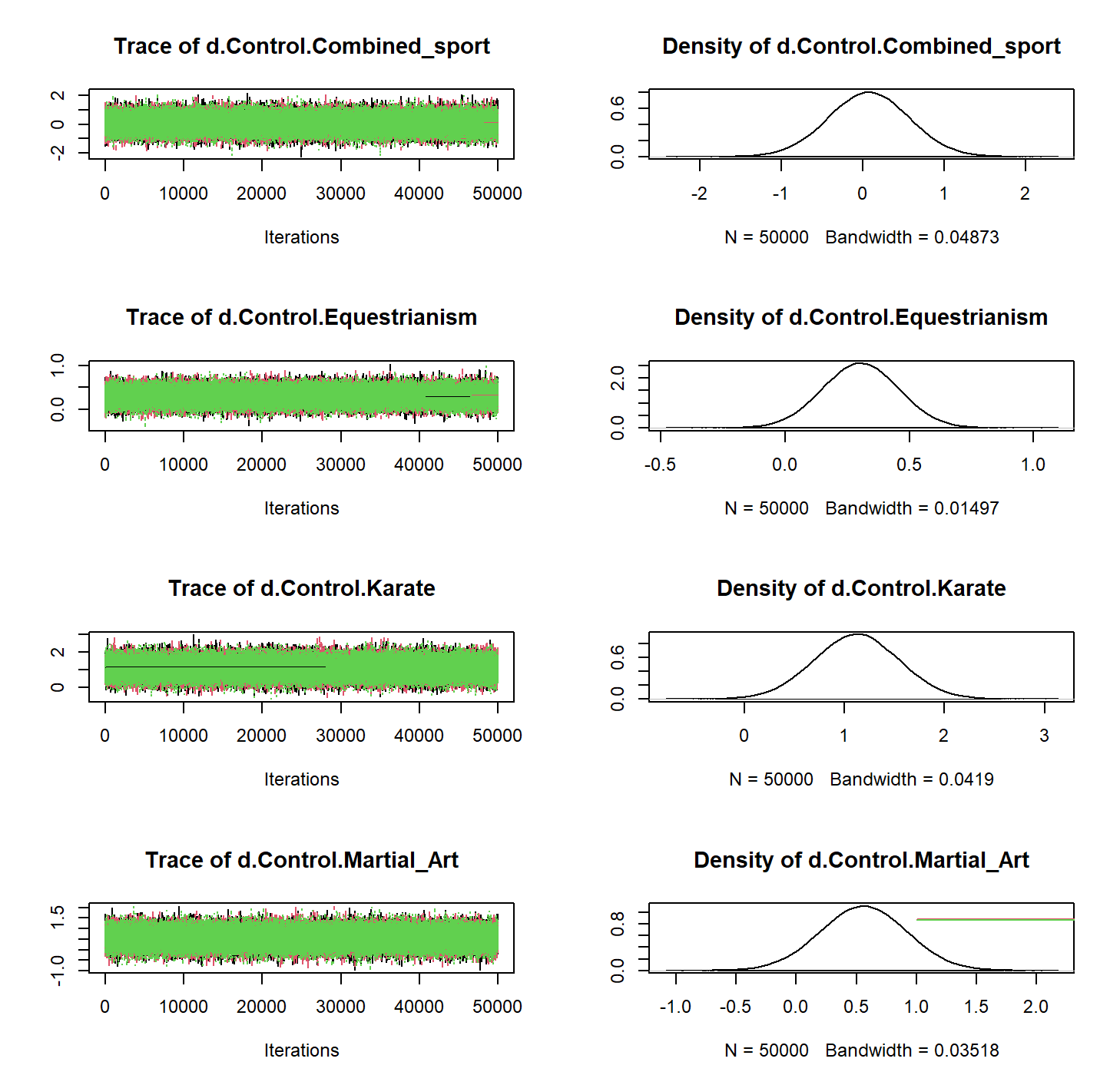

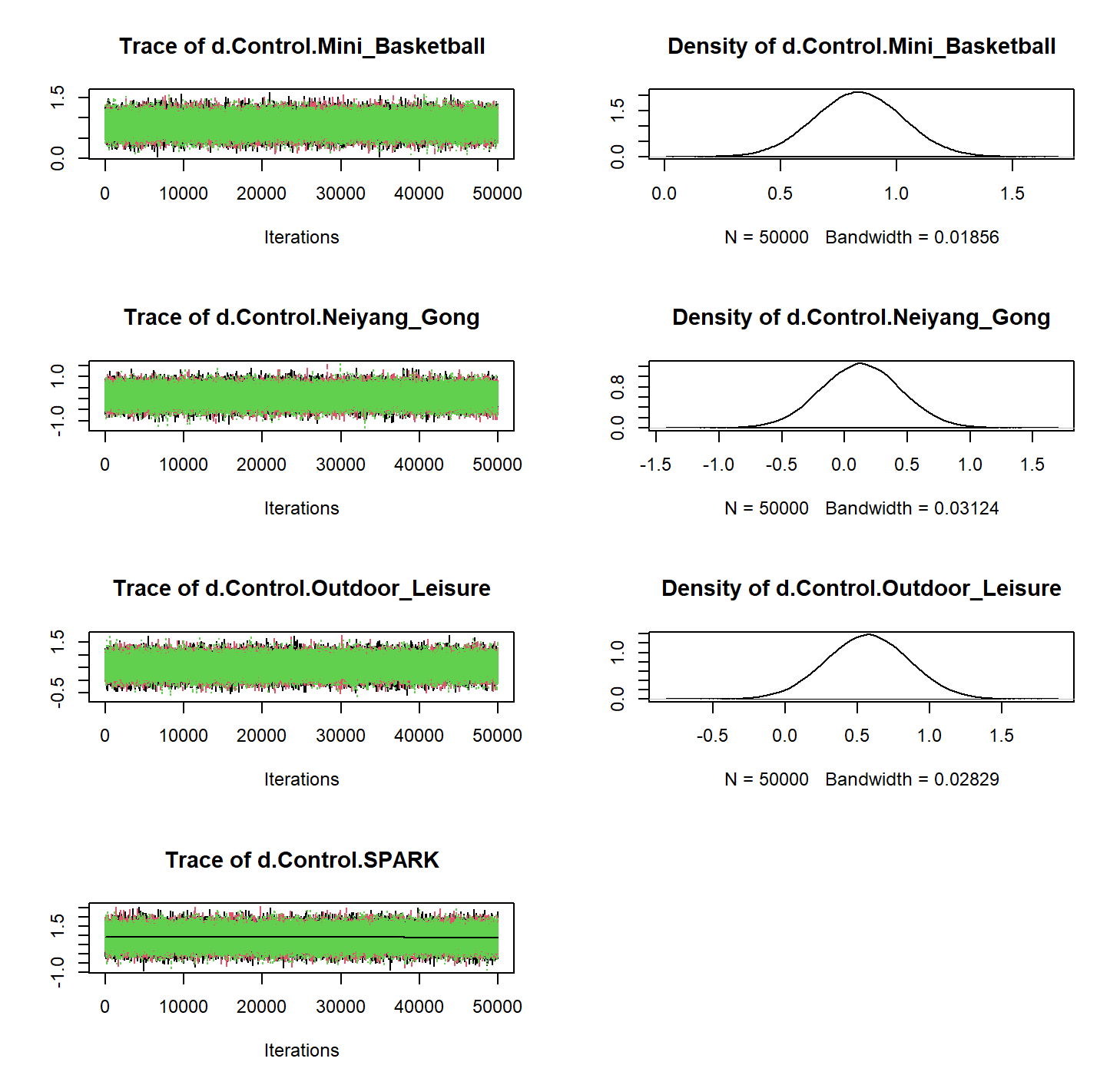


**Figure 3** Consistency model and inconsistency model


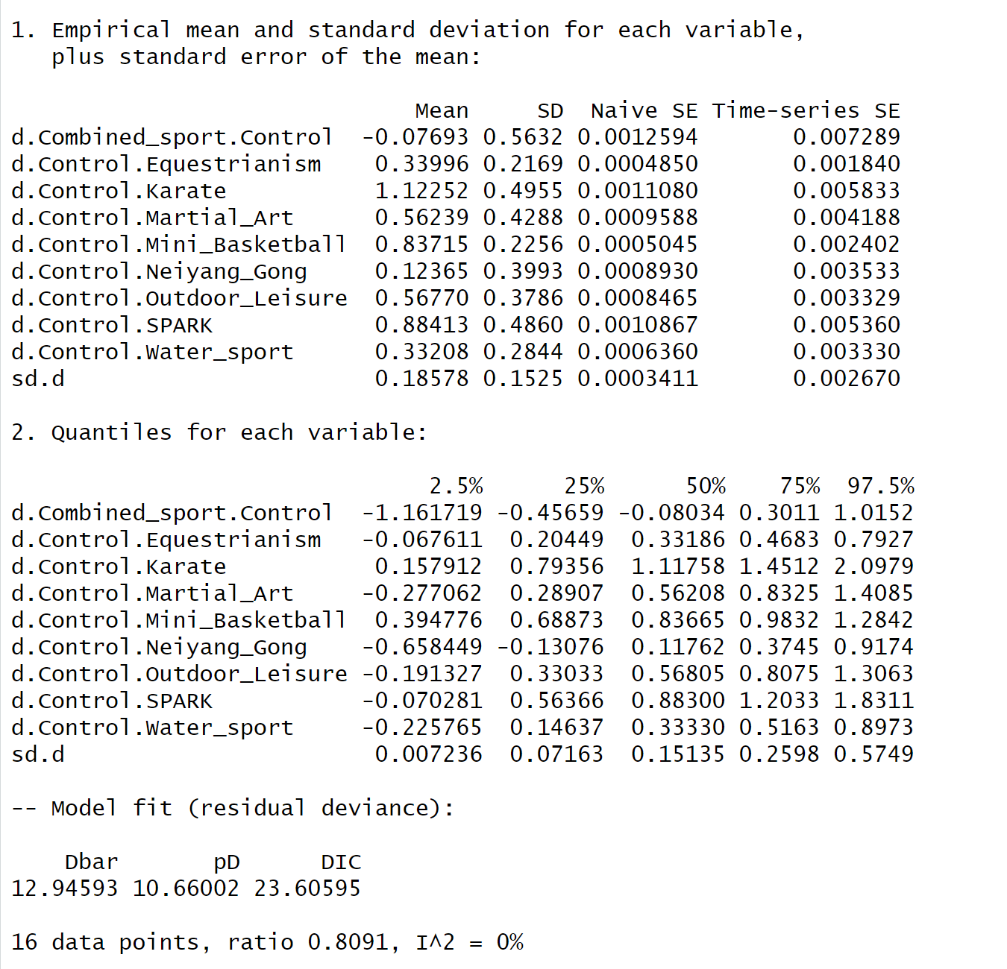

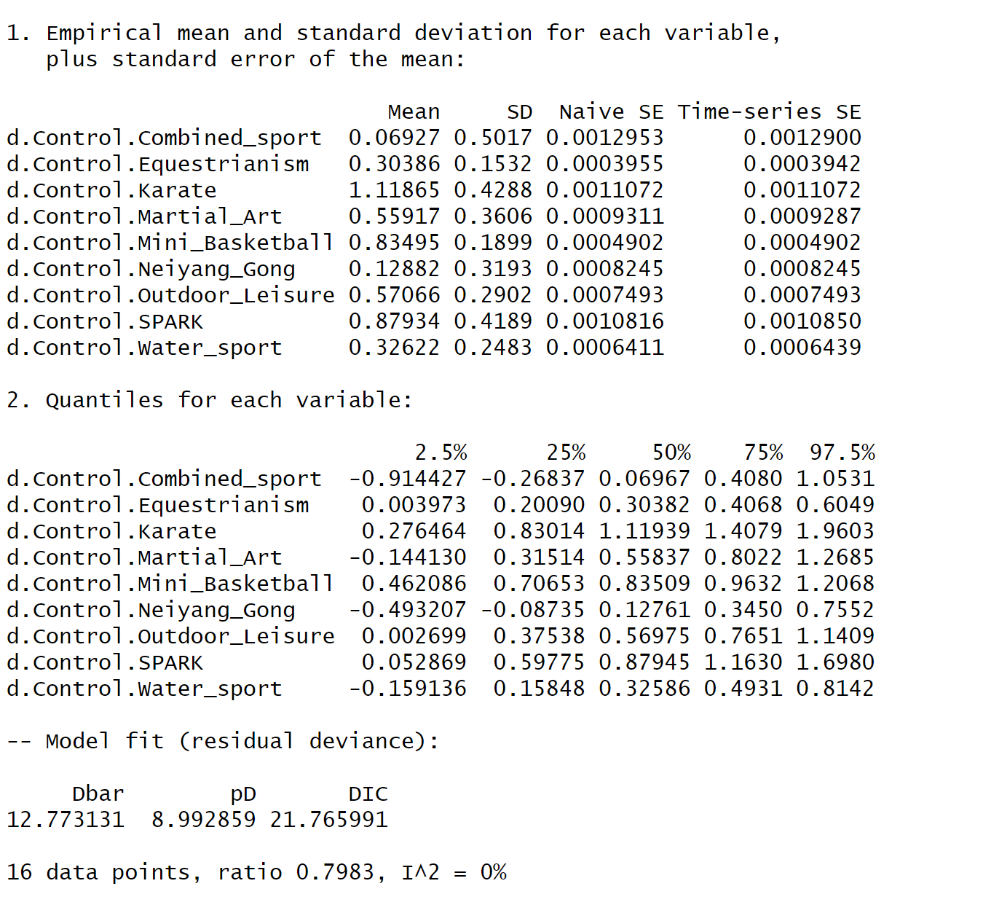


**Figure 4** Cumulative Probability


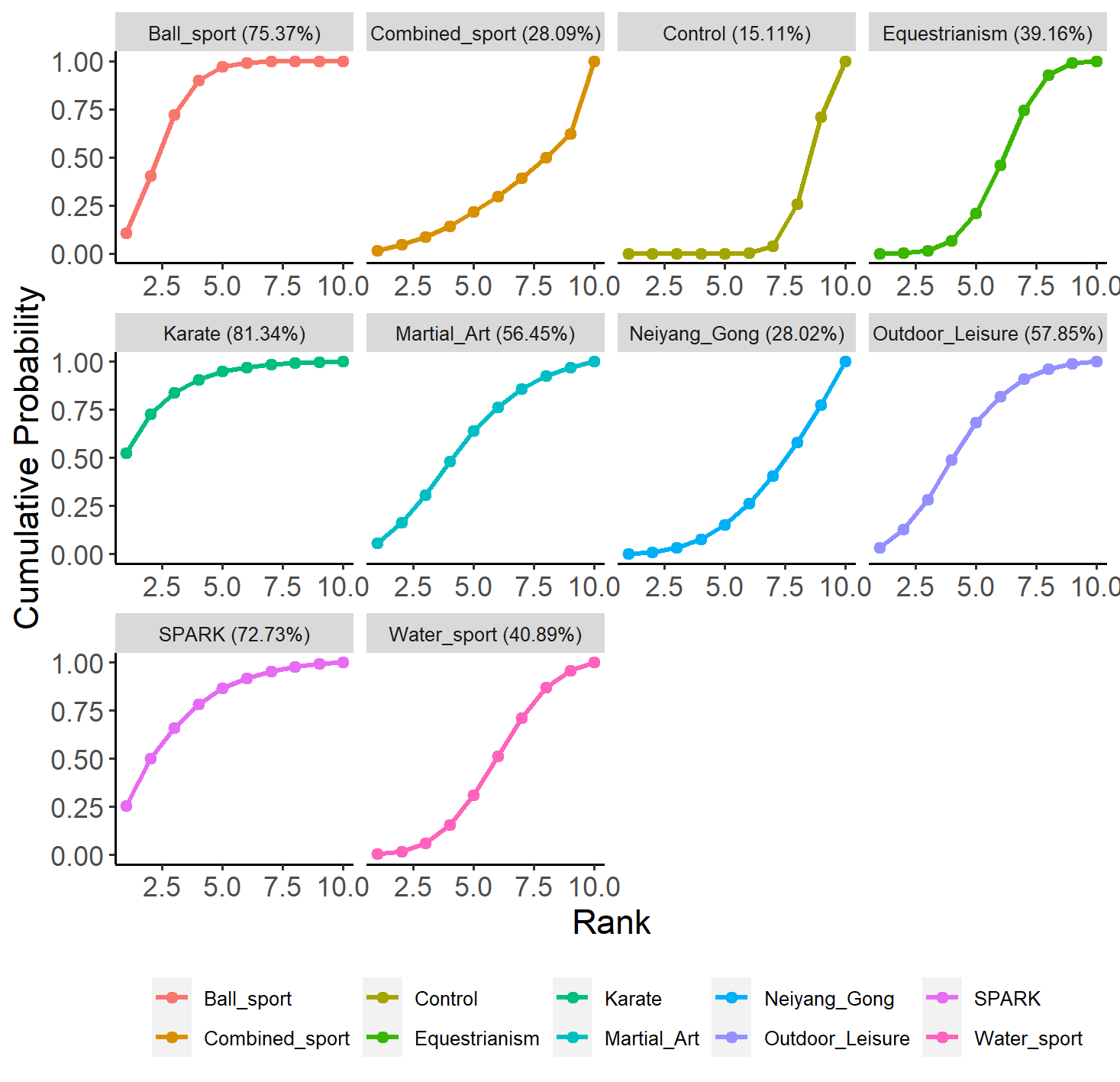


**Figure 5** Funnel plots of publication bias for outcome measures of included studies


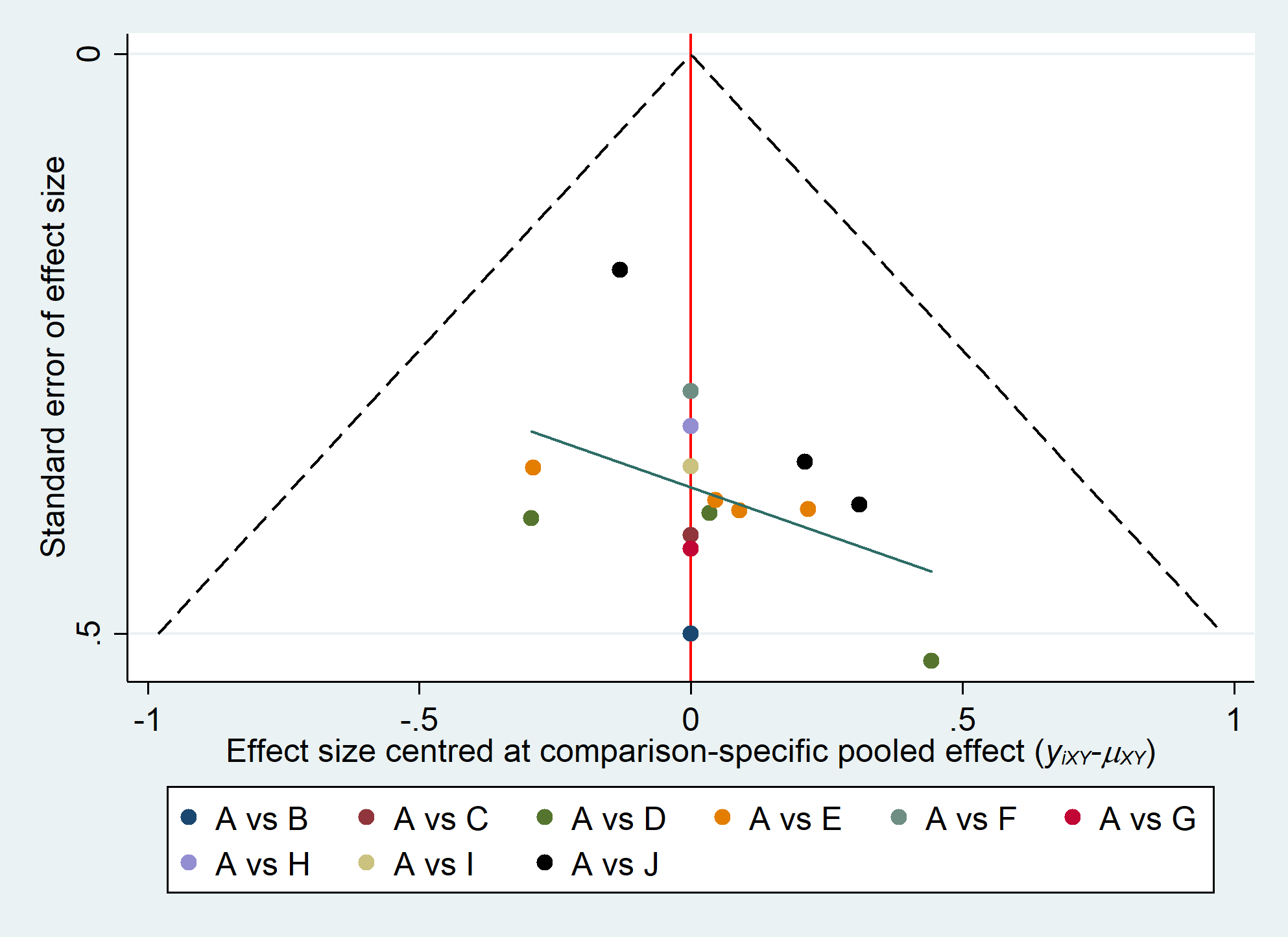


**Figure 6** Regressive analysis


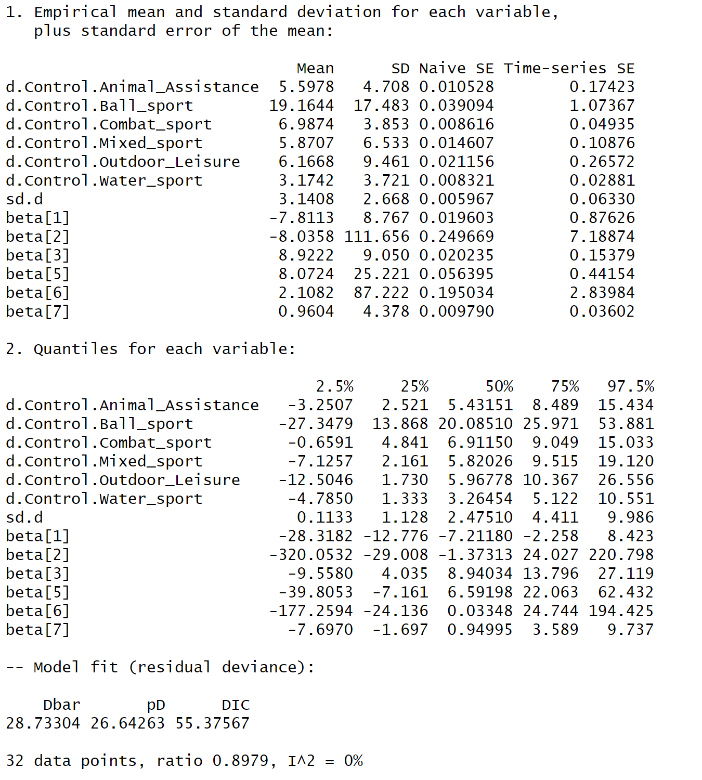

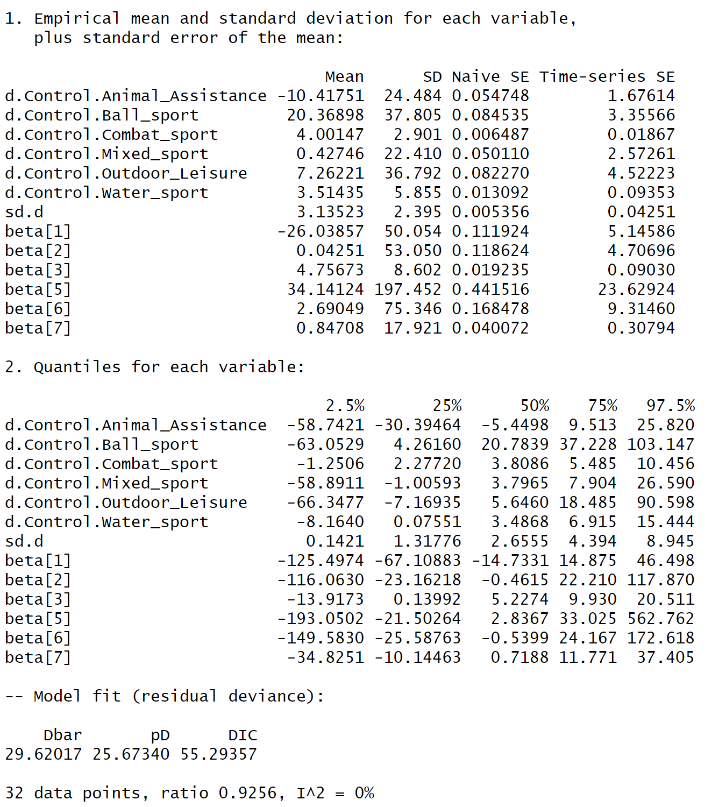


Week Times


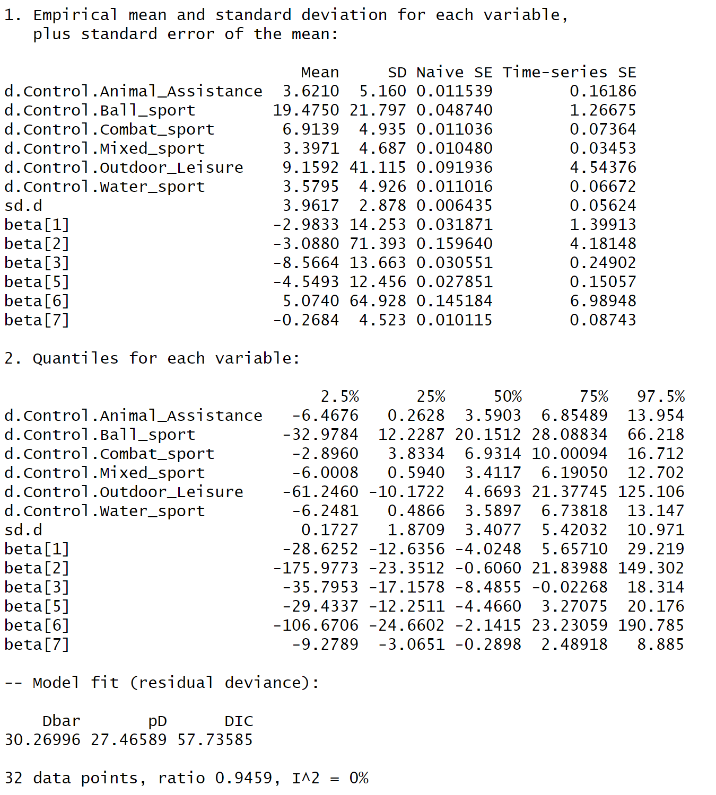

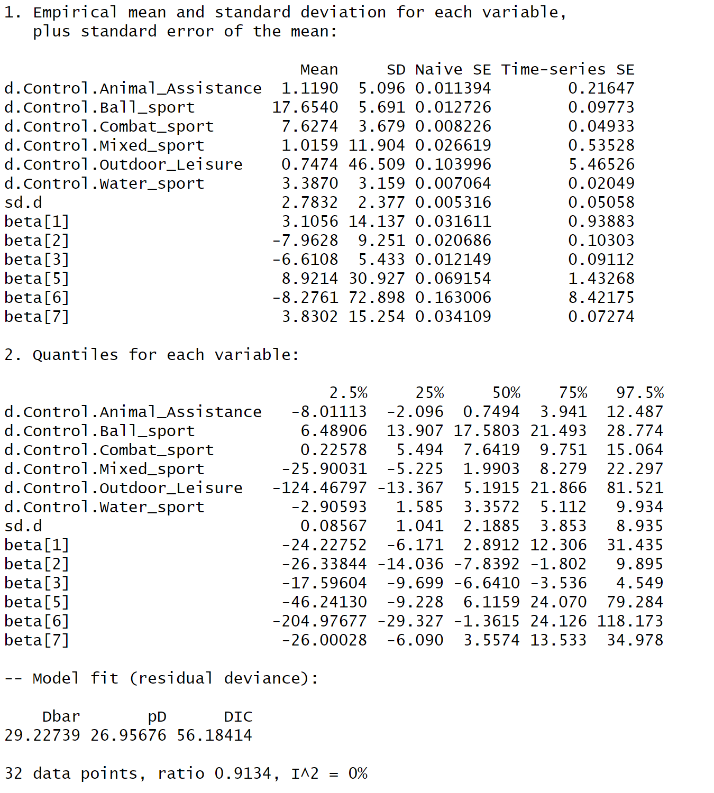


Min Total duration

**Figure 7** Regressive analysis


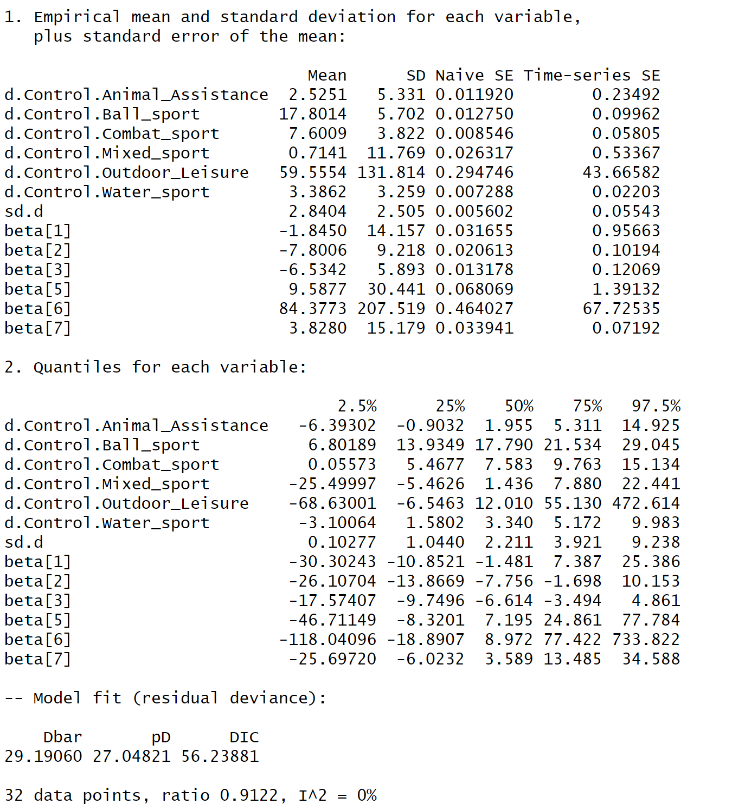

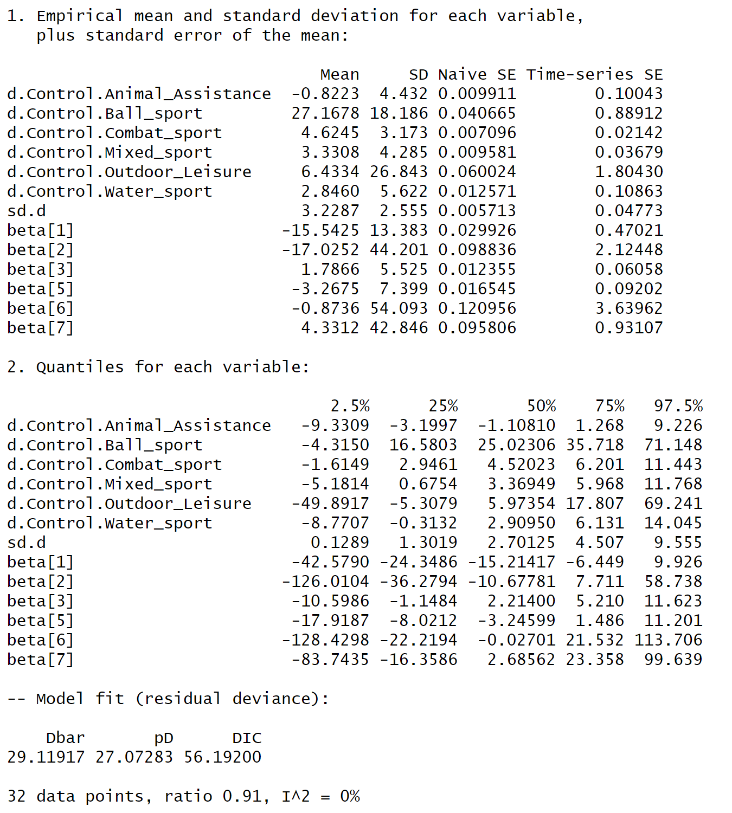


Age Year


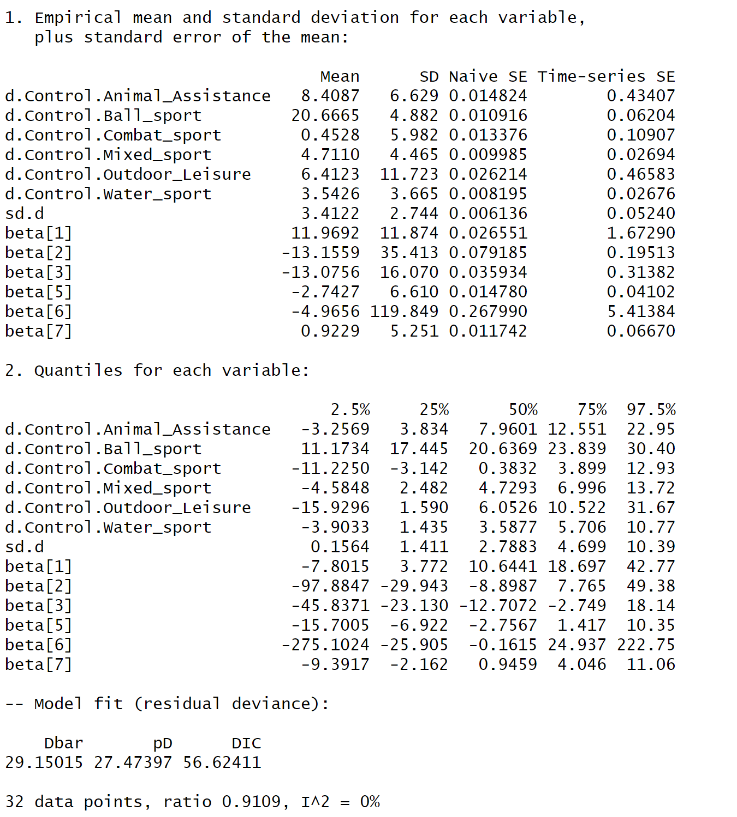

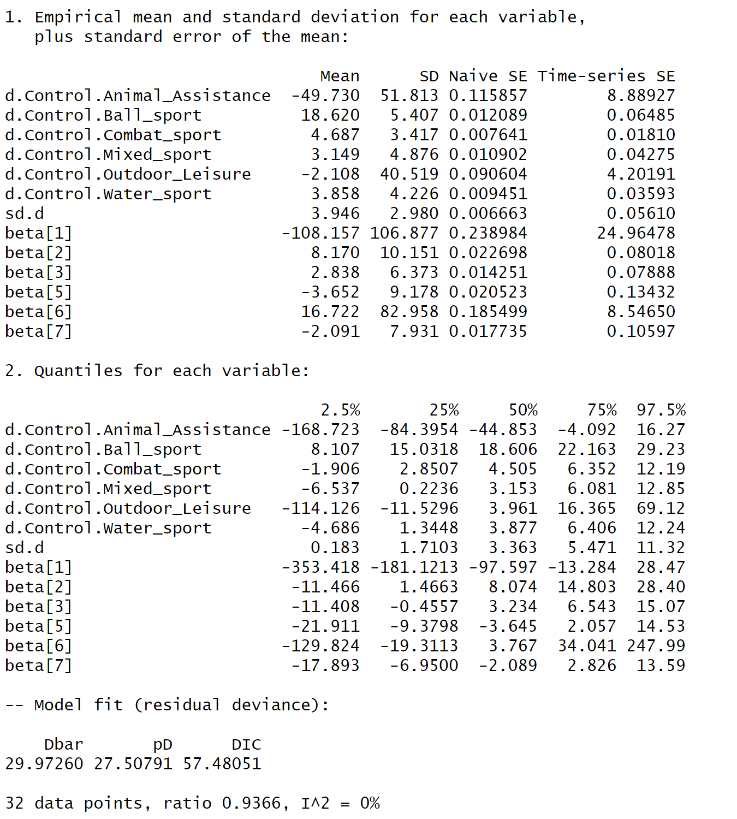


Sex Sports form
